# Genetic variation in the *CLOCK* gene is associated with idiopathic recurrent spontaneous abortion

**DOI:** 10.1371/journal.pone.0196345

**Published:** 2018-05-16

**Authors:** Alenka Hodžić, Polona Lavtar, Momčilo Ristanović, Ivana Novaković, Jelena Dotlić, Borut Peterlin

**Affiliations:** 1 Clinical Institute of Medical Genetics, University Medical Centre Ljubljana, Ljubljana, Slovenia; 2 Institute of Human Genetics, Medical Faculty, University of Belgrade, Belgrade, Serbia; 3 Clinic for Obstetrics and Gynecology, Clinical Center of Serbia, Faculty of Medicine, University of Belgrade, Belgrade, Serbia; Xavier Bichat Medical School, INSERM-CNRS - Université Paris Diderot, FRANCE

## Abstract

Physiological studies in animals and human support an important role of circadian system in reproduction. The aim of this study was to investigate the potential association of *CLOCK* gene polymorphisms with idiopathic recurrent spontaneous abortion (IRSA). We performed a case-control study. The study group consisted of 268 women with a history of three or more idiopathic recurrent spontaneous abortions and 284 women with at least two live births and no history of pathologic pregnancies all from Slovenia and Serbia. Two SNPs in the *CLOCK* gene were chosen and genotyped. The results showed a statistically significant difference in genotype distribution between the two groups in the *CLOCK* gene for rs6850524 and rs11932595. Our analysis showed that G allele under dominant model (GG+GC/CC) for rs6850524 (p = 2∙10^−4^, OR = 2.28, 95%CI = 1.46–3.56) as well as G allele under dominant model (GA+AA/AA) for rs11932595 (p = 0.04, OR = 1.47, 95%CI = 1.01–2.04) might be risk factors against IRSA. Our data suggest that genetic variability in the *CLOCK* gene is associated with IRSA warranting further confirmation and mechanistic investigations.

## Introduction

Recurrent spontaneous abortion (RSA) is clinically defined as the spontaneous loss of three or more pregnancies before the 24^th^ week of gestation. It affects approximately 0.5–3% of women and can be caused by different factors [1]. However, in about 50% of the cases the cause remains unknown [2]. Genetic contribution to idiopathic RSA (IRSA) has been extensively investigated and several studies suggest that genetic variants may predispose to RSA [3, 4].

There is growing evidence that circadian rhythms influence a large variety of physiological systems including reproduction [5, 6]. Recent evidence in animals links disruption of synchronous clock activity with the pathogenesis of pregnancy complications. Repeated shifting of the light-dark cycle, which disrupts endogenous circadian timekeeping, strongly reduces pregnancy success in mice [7]. Additionally, impaired reproductive capacities were associated with night shift work in humans as well [8]. Circadian clock genes are expressed rhythmically in the gravid uterus and placental tissue, suggesting that circadian clocks contribute to the regulation of rhythms in the developing fetus [9]. Despite the evidence of the importance of circadian rhythms in reproduction in animal models, there is very limited knowledge regarding the role of the circadian system in pregnancy in humans.

Circadian rhythms are controlled by an endogenous oscillator, the circadian clock, which consist of a network of transcriptional-translational feedback loops of many genes [10]. The *CLOCK* gene represents the central node in the network and is an essential activator of downstream elements in the pathway critical to the generation of circadian rhythms [11].

Genetic variants in the *CLOCK* gene have been previously associated with reproductive abnormalities, such as irregular menstrual cycles and increased risk of endometriosis [12, 13].

Therefore, we aimed to evaluate whether there is an association between the two genetic variants in the *CLOCK* gene and idiopathic recurrent spontaneous abortions. For that purpose, we performed a case-control genetic association study of polymorphic sites in the *CLOCK* gene on a sample of patients with IRSA in comparison with sample of women with two or more live births and no history of pathologic pregnancies.

## Materials and methods

### Ethic statement

The study was approved by committees in both countries participating in the study:

Slovenian National medical ethics committee (reference number: 152/07/09) and by the Ethics committee of Clinic for Gynecology and Obstetrics Narodni front, Serbia (reference number: 24/42-2). The study was conducted according to the Declaration of Helsinki. Each participant gave written informed consent to participate in the study.

### Subjects

A total of 552 women were included in the study. 268 women from Slavic populations from Slovenia and Serbia with a history of three or more spontaneous abortions of unknown etiology before the 24^th^ week of gestation were recruited at the Clinical Institute of Medical Genetics, Department of Obstetrics and Gynecology, University Medical Center, Ljubljana, Slovenia (152 patients) and by collaborating genetic centre Institute of Human Genetics, Faculty of Medicine, University of Belgrade, Serbia (116 patients). All patients had normal karyotypes and no history of endocrine, metabolic, autoimmune or other systemic disorders, venous or arterial thrombosis, or uterine anatomic abnormalities. The control group consisted of 284 (170 from Slovenia and 114 from Serbia) women with at least two normal pregnancies, no pregnancy loss or any other pregnancy related complications.

### SNP selection and genotyping

Two tagging SNPs rs6850524 and rs11932595 in *CLOCK* gene were obtained using the Tagger algorithm, implemented in the Haploview software as described previously [14]. To maximize statistical power in this study, two SNPs with minor allele frequency values exceeding 0.05 were included in the investigated set, as described previously [15].

Genomic DNA was isolated from the peripheral blood samples using DNeasy Blood & Tissue Kit (Qiagen). Genotyping was performed with KBioscience KASPar FRET based system (version 4.0, 2011) and predesigned SNP Genotyping Assays (KBioscience). The reactions were performed on ABI 7000 genetic analyzer using 5-40ng/μl of genomic DNA, 0.11 μl of assay and 4 μl KASP 2X Reaction Mix per reaction, following instructions in the KASP SNP Genotyping Manual (version 4.0), and with the reaction conditions specified by the manufacturer.

The allelic discrimination analysis was performed using SDS Software Version 1.2 (Applied Biosystems, Foster City, CA, USA). Genotype assignment was performed and interpreted independently by two investigators.

The STREGA guidelines were followed throughout this study [16].

### Statistical analyses

Significances of associations between allelic and genotype frequencies and disease status were analyzed using the Chi-square test (x^2^). Odds ratios (OR) and their respective 95% confidence intervals (CI) were also calculated to compare allelic and genotype distribution in patients and control subjects. Analyses were performed using the R statistical language (R 2.15.0 for Windows) x^2^ goodness-of-fit tests for deviation of genotype distribution from those predicted by Hardy-Weinberg equilibrium were also calculated, providing an additional quality control step of genotyping process. The investigated associations were regarded as significant when they reached p≤0.05. For power calculations, the pbsize2 function belonging to the gap package for R was utilized, which allows accurate power estimations under a variety of disease inheritance models (available at web address: http://cran.r-project.org/web/packages/gap/index.html).

## Results

Genotype frequencies of investigated polymorphisms were in accordance with those predicted by the Hardy-Weinberg equilibrium in the study and in the control group (p<0.05). Genotype distribution of the *CLOCK* polymorphisms of the 268 IRSA patients and 284 controls are shown in Table 1.

**Table 1:** Genotype distribution of the *CLOCK* polymorphisms of the 268 IRSA patients and 284 controls.

rs6850524								
	Genotypes						Genotype frequency (2x3 table)	
	IRSA			Controls			X^2^	p
	CC	CG	GG	CC	CG	GG		
All	35	147	86	71	117	90	15.55	4∙10–4
Slovenia	17	91	44	46	59	59		
Serbia	18	56	42	25	58	31		
rs11932595								
	Genotypes						Genotype frequency (2x3 table)	
	IRSA			Controls			X^2^	p
	AA	AG	GG	AA	AG	GG		
All	65	164	36	91	143	47	6.76	0.03
Slovenia	33	104	15	52	86	30		
Serbia	32	60	21	39	57	17		

We found statistically significant differences in genotype distribution of rs6850524 (p = 4∙10^−4^, x^2^ = 15.55) and rs11932595 (p = 0.03, x^2^ = 6.76) in the *CLOCK* gene. Our analysis showed that G allele under dominant model (GG+GC/CC) for rs6850524 (p = 2∙10^−4^, x^2^ = 13.58, OR = 2.28, 95%CI = 1.46–3.56) as well as G allele under dominant model (GG+GA/AA) for rs11932595 (p = 0.04, x^2^ = 4.12 OR = 1.47, 95%CI = 1.01–2.04) might be risk factors against IRSA.

The statistical power to detect a significant result in the presence of the actual genotype-phenotype effect with genotype relative risk equal to at least 1.4, was 82%, when taking into account the sample size, the significance threshold of 0.05, the prevalence of IRSA in the general population, disease allele frequency and considering multiplicative model of genetic association.

## Discussion

Progress in understanding the pathological mechanisms underlying recurrent spontaneous abortions has been greatly hampered by the complex nature of the disorder. Previous studies suggest that genetic factors play an important role in unexplained recurrent spontaneous abortions [3, 4]. In the case-control study we found evidence of an association between IRSA and gene variants of the *CLOCK* gene in a sample of 552 women. To the best of our knowledge, this is the first association study between genetic variability in the *CLOCK* gene and IRSA.

Several lines of evidence support our findings. In humans, it has been shown that night shift workers have an increased rate of reproductive abnormalities as well as adverse pregnancy outcomes in terms of miscarriage, low birth weight and preterm birth [17]. Similarly it has been seen in two other studies, where night shift work had influence on early spontaneous pregnancy loss, time to achieve pregnancy, menstrual cycle disturbances and endometriosis [18, 19]. Circadian disruption as potential reproductive hazard has been also noticed in flight attendants; where risk of a first-trimester miscarriage was increased in women flying during home-base sleep hours [20].

Genetic variants in the circadian rhythm genes ARNTL and NPAS2 have been suggested to contribute to fertility, and the genetic variants in the ARNTL gene has been associated with a higher number of miscarriages. Certain genotypes in the Npas2 gene have been associated with a decreased number of miscarriages [21]. Genetic variation in circadian rhythm genes ARNTL2, CRY2, DEC1, PER3 and RORA have also been associated with increased risk of placental abruption [22].

Furthermore, it has been demonstrated that low *CLOCK* expression level in pregnant women may lead to spontaneous abortion [23]. Significantly lower *CLOCK* gene expression has been detected in spontaneously aborted fetuses compared with fetuses from induced abortion [23].

In mice, mutations in the circadian *CLOCK* gene cause multiple reproductive abnormalities, including increased fetal reabsorption during pregnancy and an elevated rate of full-term pregnancy failure [24, 25].

Although functional studies on rs6850524 and rs11932595 were not performed in IRSA, the polymorphisms affect the expression of the *CLOCK* gene in a number of different tissues including nervous system, subcutaneous adipose tissue, skin, muscle, esophagus, thyroid (https://www.gtexportal.org/home/snp/rs6850524, https://www.gtexportal.org/home/snp/rs11932595).

However, this study has limitations. A relatively small sample might influence the statistical power and, consequently, the detection of the true effect size. Also, genetic association studies are prone to α (Type I) statistical error and population-specific genotype effects.

In conclusion, we provide evidence that genetic variants in the *CLOCK* gene might be associated with IRSA. Further confirmation and mechanistic investigation of circadian system in idiopathic recurrent spontaneous abortion is warranted.
